# Discovery of os cordis in the cardiac skeleton of chimpanzees (*Pan troglodytes*)

**DOI:** 10.1038/s41598-020-66345-7

**Published:** 2020-06-10

**Authors:** Sophie Moittié, Kerstin Baiker, Victoria Strong, Emma Cousins, Kate White, Mátyás Liptovszky, Sharon Redrobe, Aziza Alibhai, Craig J. Sturrock, Catrin Sian Rutland

**Affiliations:** 1 School of Veterinary Medicine and Science, Faculty of Medicine and Health Science, University of Nottingham, Leicestershire, LE12 5RD UK; 2 Twycross Zoo, East Midland Zoological Society, Atherstone, CV93PX UK; 3 Present Address: School of Animal, Rural and Environmental Sciences, Nottingham Trent University, Brackenhurst Campus, Southwell, Nottinghamshire NG25 0QF UK; 4 School of Biosciences, University of Nottingham, Leicestershire, LE12 5RD UK

**Keywords:** Developmental biology, Zoology

## Abstract

Cardiovascular diseases, especially idiopathic myocardial fibrosis, is one of the most significant causes of morbidity and mortality in captive great apes. This study compared the structure and morphology of 16 hearts from chimpanzees (*Pan troglodytes*) which were either healthy or affected by myocardial fibrosis using X-ray microtomography. In four hearts, a single, hyperdense structure was detected within the right fibrous trigone of the cardiac skeleton. High resolution scans and histopathology revealed trabecular bones in two cases, hyaline cartilage in another case and a focus of mineralised fibro-cartilaginous metaplasia with endochondral ossification in the last case. Four other animals presented with multiple foci of ectopic calcification within the walls of the great vessels. All hearts affected by marked myocardial fibrosis presented with bone or cartilage formation, and increased collagen levels in tissues adjacent to the bone/cartilage, while unaffected hearts did not present with os cordis or cartilago cordis. The presence of an os cordis has been described in some ruminants, camelids, and otters, but never in great apes. This novel research indicates that an os cordis and cartilago cordis is present in some chimpanzees, particularly those affected by myocardial fibrosis, and could influence the risk of cardiac arrhythmias and sudden death.

## Introduction

Wild chimpanzees are classified as endangered by the International Union for Conservation of Nature (IUCN)^1^. Threats include habitat conversion and fragmentation, poaching, and infectious diseases mostly due to anthropozoonotic disease outbreaks. Maintaining healthy captive populations is a fundamental part of *ex-situ* conservation programmes, and the knowledge of detailed anatomy and physiology as well as epidemiology and pathophysiology of diseases affecting great apes are crucial tools in enhancing their health and reducing mortality. Cardiovascular disease (CVD) is associated with significant proportional mortality in captive great apes^2–6^. Diseases of the cardiovascular system have been reported to affect 77% of adult chimpanzees that died between 1990 and 2003 in zoos accredited by the American Association of Zoo and Aquaria^7^. Whereas in humans coronary artery disease is the most frequent type of heart disease, in chimpanzees the most common entity described is idiopathic myocardial fibrosis (IMF)^8^. Also referred to as fibrosing cardiomyopathy or interstitial myocardial fibrosis, this lesion is characterised by an accumulation of fibrous connective tissue and is associated with impaired cardiac function and sudden cardiac death^4–6,9^. The aetiopathogenesis of idiopathic myocardial fibrosis (IMF) in great apes is poorly understood. Histological examination is the definitive means by which the degree of cardiac fibrosis can be assessed in both human and veterinary medicine^10^. In humans, endomyocardial biopsies can be performed for the investigation of some cardiomyopathies or unexplained arrhythmias. This procedure is not performed in nonhuman hominids due to its invasive nature and the unknown sensitivity and specificity as a diagnostic test in these species. All confirmed cases of IMF in chimpanzees have been diagnosed via post-mortem histopathological examination. Lesions vary from minimal to marked (frequently chronic) and show variable degree of diffuse, reactive interstitial fibrosis and/or focally extensive or multifocal areas of replacement fibrosis.

Cardiac imaging is often performed for the assessment of CVD in animals and humans. Modalities commonly used in human medicine include radiography, echocardiography, computed tomography (CT), and cardiac magnetic resonance imaging (MRI) with or without contrast agents. In zoological medicine, however, most of the advanced techniques are not readily available and radiography and ultrasonography remain the most widely used techniques. Despite being a clinical challenge, echocardiographic parameters of clinically normal but anaesthetised chimpanzees have been published^11^. Anaesthesia is normally required for these animals but makes interpretation more complicated in many cases. Although echocardiography is useful to detect cardiac structural changes and function, myocardial fibrosis can occur with only subtle image variations^12^, and the effect of anaesthetic agents complicates the interpretation of echocardiographic findings. With the exception of an anatomical study comparing cardiac innervation and position in orang-utans, chimpanzees, and gorillas, no reports exist on the use of CT scanning to assess cardiac function or structure in great apes^13^.

X-ray microtomography (microCT), like CT, is a non-destructive X-ray imaging technique that produces three-dimensional images from two-dimensional trans-axial projections. MicroCT generally works on smaller specimens but with a much higher resolution than standard CT producing images and maps with voxels in the micrometre range. MicroCT has been used since the 1980’s in the industrial and biomedical sectors. Its main applications in the latter sector are studies involving bone structure and function, the development of tissue engineering techniques and for the detection of vascular and soft tissue lesions when combined with the use of contrast agents^14^. Whereas CT is widely used in clinical studies and routine diagnostics and treatment, in veterinary science, microCT has mostly been used in anatomical research studies and for use in cadaver materials^15,16^. Its application in published clinical practice in living animals has been restricted due to high radiation doses but the detection of bone fractures and oral diseases in rabbits and the study of diseases mechanisms in laboratory animals have been undertaken^17–19^.

As IMF has been linked to the occurrence of cardiac arrhythmias and sudden death in chimpanzees^4,5^, the study of the anatomy and structure of the cardiac conduction system and its surrounding tissues is of utmost importance. In all mammals, the electrical impulse for each cardiac cycle starts in the sino-atrial node, situated in the right atrium. Depolarisation spreads through the atrial muscle cells and reaches the atrio-ventricular (AV) node, located anterior to the coronary sinus ostium and directly superior to the septal leaflet of the tricuspid valve^20^. The Bundle of His passes the electrical impulse to the infranodal conduction system through the cardiac skeleton.

The fibrous skeleton of the heart is a supportive and functional structure formed by fibrous rings of high-density connective tissue (annulus fibrosus) surrounding the two atrioventricular orifices (also called the atrioventricular rings), the aortic orifice (frequently termed the aortic ring) and opening of the pulmonary trunk (pulmonary ring)^21–23^. Areas connecting these fibrous rings form the central fibrous body and right and left fibrous trigones or trigonum fibrosum dextrum et sinistrum, which in certain species contain fibrocartilage, hyaline cartilage (cartilago cordis) and even bone (os cordis)^24^. The cardiac skeleton provides rigidity to prevent dilatation of valves and outflow tracts, gives attachments to the valves leaflets and myocardium, and electrically isolates the atria from the ventricles^23^. The presence of an os cordis is a regular finding in large ruminants such as cattle, ox, water buffalos and sheep and has also been described in otters and camelids^25–29^, but is not reported in other mammals. In cattle, the os cordis is located near the junction of the interatrial and interventricular septa and extends anteriomedially into the right atrioventricular ring. Occasionally, a second bone is present within the left atrioventricular ring^27^. Although the exact localisation, size and number of the os cordis varies, in all species it lies within the trigonum fiobrosum, adjacent to the AV node and consists of trabecular bone with marrow and fat. Its function is unclear but it is believed to serve as a pivot and anchoring support for the cardiac valves^27,30^. Cartilage (cartilago cordis) can also be present within the cardiac skeleton of individuals of other animal species such as horses, pigs, canids, felids, mice, rats, snakes, white rhinoceros and Syrian hamsters^24,25,31–33^. In humans, mineralisation of the cardiac skeleton can occur as mitral and aortic annular calcification and aortic valve sclerosis. They are considered degenerative changes associated with aging and are linked with cardiovascular disease^34^.

The aim of this study was to characterise the hyperdense tissue discovered in the chimpanzee heart using microCT and histopathological techniques. In addition, the relation between the presence of os cordis, cartilago cordis or foci of ectopic calcifications and the level of IMF, age and sex of the animals were studied.

## Results

### Initial cardiac examination and interstitial fibrosis histopathology

Three of the 16 investigated hearts, S2, S6 and S9, showed no histological evidence of IMF. Three chimpanzees were affected by marked IMF (IMF level = 6): two males aged 22 and 37 years (S5 and S8 respectively) and one female aged 25 years (S10). The remaining ten animals were affected by minimal to moderate levels of IMF (IMF levels ranging from 1 to 5; Table 1). None of the chimpanzees had known cardiac disease before death. Clinical and pathological results are summarised in Table 1 and Supplementary Table 1.

**Table 1:** Chimpanzee sex, age, heart weight, IMF levels and microCT and histological observations presented in age order for each sex. IMF levels are ranked from 0 (no IMF) to 6 (marked IMF).

Specimen ID	Sex	Age	Heart weight (g)	IMF level	CT scan findings (confirmed histologically)
S1	Male	10	299	2	-
S2	Male	11	201	0	-
S3	Male	19	396	1	-
S4	Male	20	378	1	-
S5	Male	22	709	6	Trabecular bone within cardiac skeleton
S6	Male	22	362	0	-
S7	Male	28	485	2	-
S8	Male	37	446	6	Cartilage within cardiac skeleton
S9	Female	21	253	0	-
S10	Female	25	455	6	Trabecular bone within cardiac skeleton
S11	Female	32	276	2	-
S12	Female	32	242	1	Multiple foci of ectopic calcification
S13	Female	42	422	5	Multiple foci of ectopic calcification
S14	Female	46	324	5	Endochondral ossification within cardiac skeleton + Multiple foci of ectopic calcification
S15	Female	47	325	3	-
S16	Female	59	551	3	Multiple foci of ectopic calcification

### X-ray microtomography

**Figure 1:**
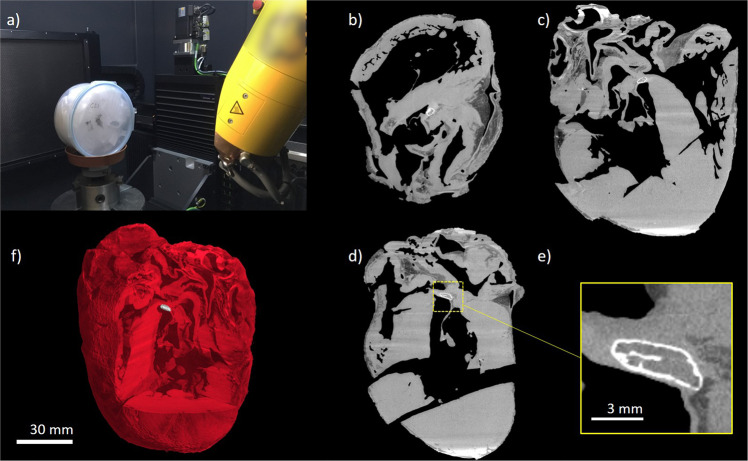
MicroCT images of showing hyperdense structures within the cardiac skeleton of S3. Data was visualised using VGStudioMAX v2.2 Software, https://www.volumegraphics.com/en/products/vgstudio-max.

X-ray microCT generated clear images of the structure of the formalin fixed hearts (Fig. 1). The dissection cuts made during a detailed macroscopic examination and sampling for histopathology were apparent on the scans. Cardiac chambers appeared as hypodense spaces (corresponding to darker regions on images, Fig. 1), whilst cardiac muscle, valves and vascular walls contrasted well at a medium density. Tissue in these areas was homogeneous overall, therefore it was noted that histomorphological changes in the formalin-fixed parenchyma such as myocardial fibrosis could not be observed using the parameters set for microCT scanning.

**Figure 2:**
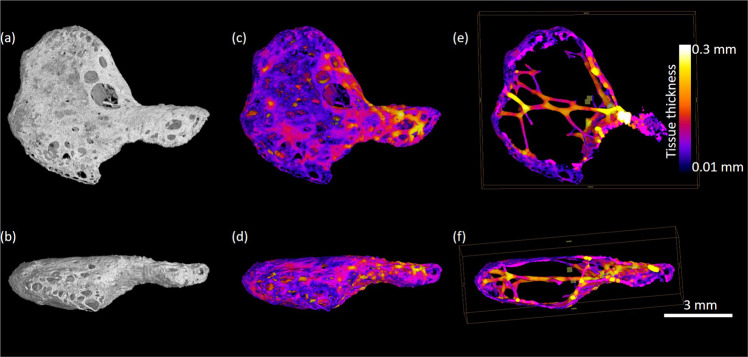
High-resolution microCT images of dissected hyperdense structure; *os cordis* from specimen 5. Figures depict rendered specimens (a + b) and those undergoing local thickness measurement (c-f) from two different angles at different depths. Data was visualised using VGStudioMAX v2.2 Software https://www.volumegraphics.com/en/products/vgstudio-max. BoneJ plugin http://bonej.org/ and ImageJ v1.44 https://imagej.nih.gov/ij/ were used to image and quantify bone thickness.

**Figure 3:**
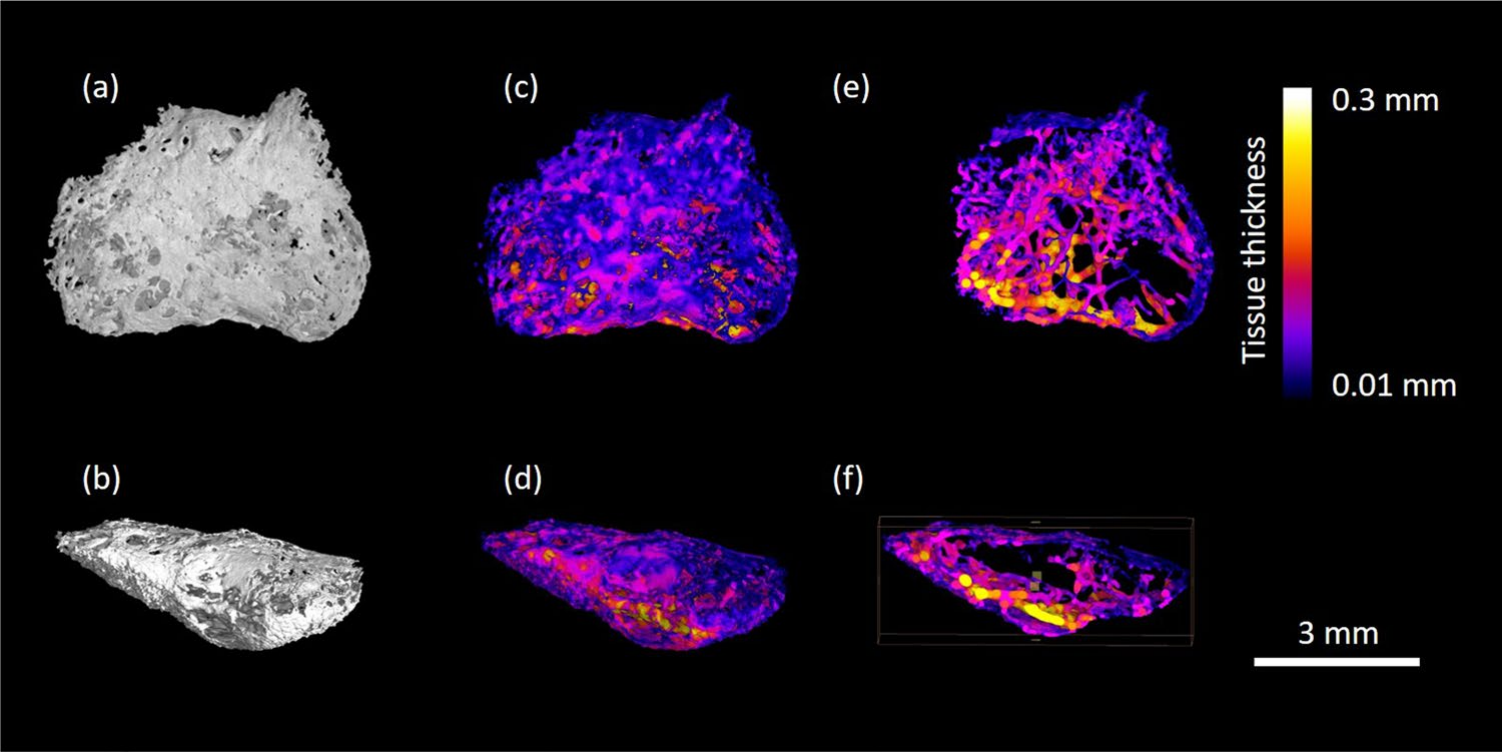
High-resolution microCT images of dissected hyperdense structure; *os cordis* from specimen 10. Figures depict rendered specimens (a + b) and those undergoing local thickness measurement (c-f) from two different angles at different depths. Data was visualised using VGStudioMAX v2.2 Software https://www.volumegraphics.com/en/products/vgstudio-max. BoneJ plugin http://bonej.org/ and ImageJ v1.44 https://imagej.nih.gov/ij/ were used to image and quantify bone thickness.

**Figure 4:**
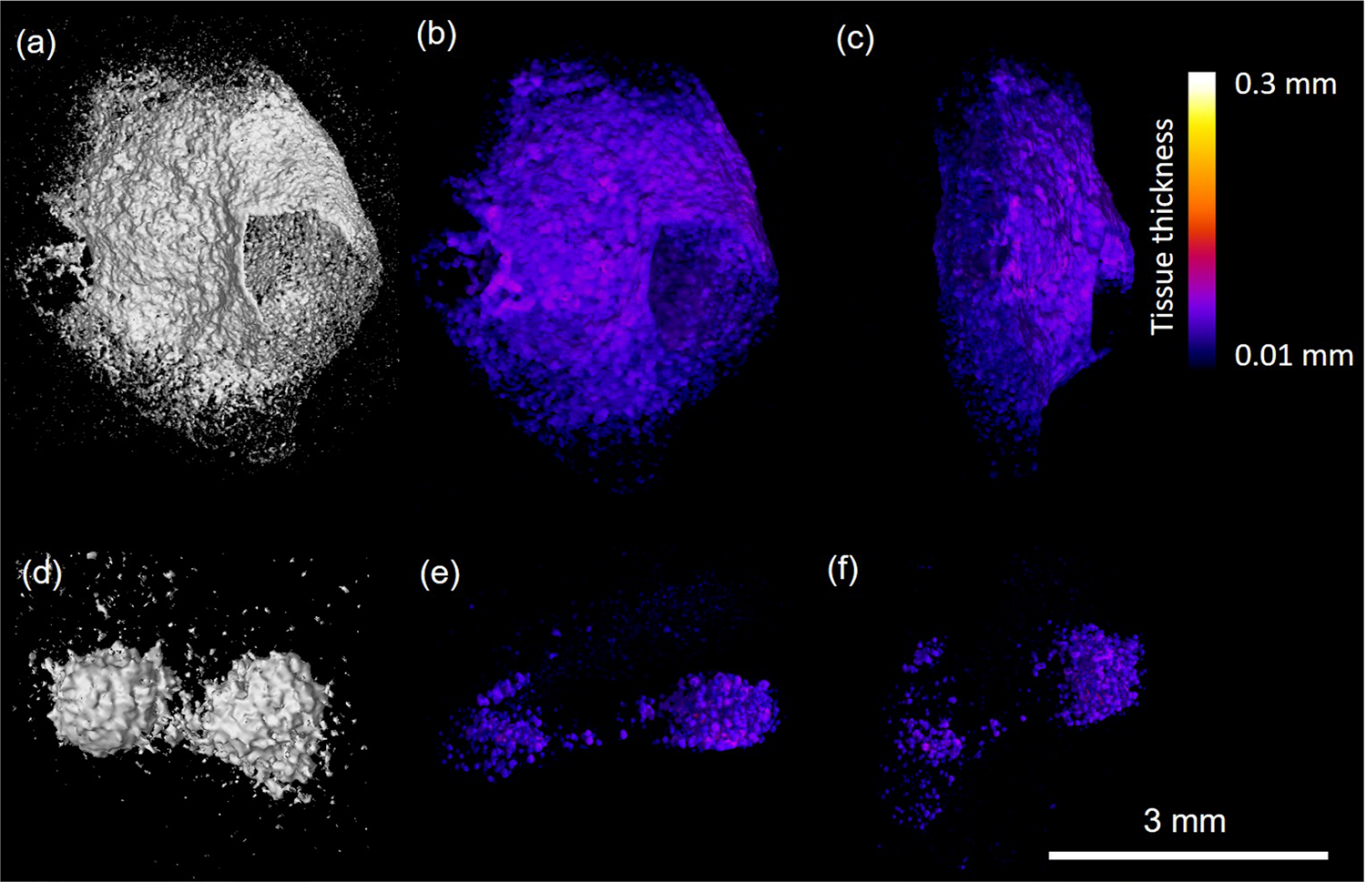
High-resolution microCT images of dissected hyperdense structure. a-c) os cordis from specimen S14 and d-f) cartilago cordis from specimen 8. Figures depict rendered specimens (a + d) and those undergoing local thickness measurement (b,c,e,f). Data was visualised using VGStudioMAX v2.2 Software https://www.volumegraphics.com/en/products/vgstudio-max. BoneJ plugin http://bonej.org/ and ImageJ v1.44 https://imagej.nih.gov/ij/ were used to image and quantify bone thickness.

Nine hearts showed no hyperdense areas. These hearts had either IMF levels of 0 (n = 3), 1 (n = 2), 2 (n = 3) or 3 (n = 1; Table 1). In the remaining seven hearts, hyperdense areas were detected and were compatible with areas of mineralisation or bone formation (shown as very bright regions in the images). These hearts had IMF levels of 1 (n = 1), 3 (n = 1), 5 (n = 2) or 6 (n = 3). In four of these specimens (S5, S8, S10, S14, all IMF levels 5 or 6), a hyperdense structure was located within the cardiac skeleton, on the valvular plane between the mitral and tricuspid valve (right fibrous trigone), just above the interventricular septum (Figs. 2–4 and Supplementary Video 1 showing a microCT scan of S10). Higher-resolution images of these structures revealed trabecular bone in S5 (Fig. 2) and S10 (Fig. 3), non-trabecular bone in S14 (Fig. 4a–c) and a mineralised structure in S8 (Fig. 4d–f) later confirmed as cartilage via histological examination. The measurements of these features were taken following microCT analysis (Table 2). S5 was shown to have the largest volume, surface area and tissue thickness compared to the other structures.

**Table 2:** Dimensions of os cartilago and os cordis features within the hearts. SD = standard deviation.

Specimen ID	Length (mm)	Width (mm)	Depth (mm)	High density material volume (mm^3^)	Mean local thickness (mm ± SD)	Maximum local tissue thickness (mm)	Surface area (mm^2^)
S8 cartilage	1.5	0.944	0.48	0.30	0.058 ± 0.033	0.146	7.659
S5 bone	7.4	7.6	4.2	9.37	0.147 ± 0.064	0.437	218.10
S10 bone	5.5	2.6	5	4.25	0.094 ± 0.047	0.234	162.44
S14 bone	5.5	7.8	5.9	1.73	0.053 ± 0.018	0.101	87.32

In S12, S13, S14 and S16, multiple, well-demarcated areas of increased density were detected mostly within the walls of the great vessels, and for S12 and S16, within the cardiac skeleton as well. These were later confirmed histologically as foci of ectopic calcification.

### Histopathology of os cordis, cartilago cordis and other hyperdense structures

**Figure 5:**
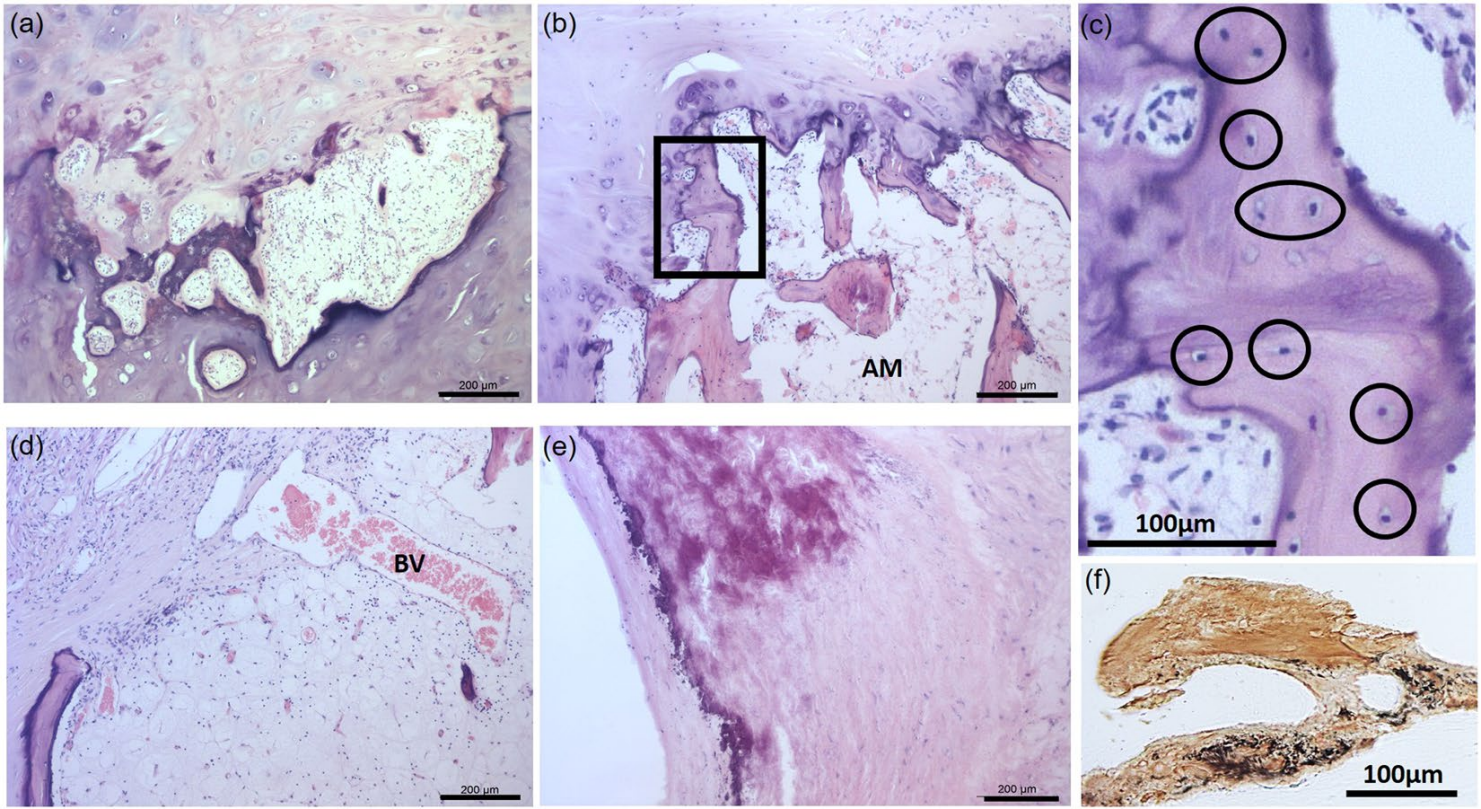
Cardiac bone and ectopic calcification histology. Areas of endochondral ossification were evident in photomicrographs of ectopic hyaline cartilage and lamellar bone with adipose marrow (AM), fibrovascular stroma, blood vessels (BV) and small numbers of haematopoietic cells in addition to osteocytes. a) S14 and b-d) S10. The box in b) is shown in a higher magnification in c) to highlight osteocytes. Hyperdense areas on microCT images revealed well-demarcated areas of ectopic calcification in some specimens shown by histology in e) tunica media of the aortic wall from S16. f) Photomicrograph of Von Kossa positive staining (brown/silver in colour) showing areas of calcification in S14. Scale bars represent 200 µm in a,b,d,e and 100 µm in c + f.

Histopathological examination was utilised in order to determine the exact nature of the hyperdense structures. Histology of S5 and S10 hyperdense structures revealed focal areas of ectopic bone tissue formation, which contained bone marrow composed of marrow adipose tissue, supportive stromal cells, numerous osteocytes and additionally in the case of S5 large numbers of haematopoietic cells including red and white blood cell precursors (Fig. 5b–d). In S14, histology of the hyperdense area within the cardiac skeleton revealed a focal area of mineralised cartilaginous metaplasia with endochondral ossification (Fig. 5a). The other hyperdense areas visible in S14 were foci of ectopic calcifications, no attributes of cartilage or bone were observed within ectopic calcifications. In S8, a focal, a well-demarcated area of hyaline cartilage development with an area of central necrosis and subsequent dystrophic mineralisation (a cartilago cordis) was observed.

In S12, S13, and S16 only areas of ectopic calcification were detected histologically. S12 presented foci of ectopic calcification in the aorta and within the cardiac skeleton. Both S13 and S16 presented foci of ectopic calcification of the aortic wall (Fig. 5e). S16 also presented with well-demarcated foci of ectopic calcification within the cardiac skeleton, between the right and left ventricular outflow tract and the interventricular septum. Von Kossa staining also highlighted calcification within hyperdense material (Fig. 5f).

### **Statistical analysis of os/cartilago cordis formation, interstitial myocardial fibrosis, age and sex**

Hearts presenting with os or cartilago cordis had significantly higher levels of IMF than hearts without os/cartilago cordis (Mann-Whitney U = 0.5, p-value=0.001). Although the level of IMF was significantly associated with age (Spearman’s rho=0.546, p-value 0.029), it was not associated with sex (Mann-Whitney U = 24.00, p-value=0.44). The presence of os/cartilago cordis was not associated with age (t = −0.483, p-value=0.636) nor with sex (Fisher’s exact test p-value>0.99). The presence of ectopic calcifications, however, was significantly associated with age (t = −3.308, p-value=0.005), with a mean age of 24.5 years (sd = 10.44) for animals without ectopic calcification and a mean age of 44.75 years (sd = 11.177) for animals with ectopic calcifications. Although the presence of ectopic calcifications was not significantly associated with sex (Fisher’s exact test p-value=0.077), half of the females presented with ectopic calcifications, while none of the males did. Heart weight was significantly associated with the level of IMF (Spearman’s rho= 0.642, p-value=0.007) but not with the presence of bone/cartilage cordis (t = −1.960, p-value = 0.07).

### **Collagen proportions in cardiac skeleton containing os/cartilago cordis or ectopic calcifications**

**Figure 6:**
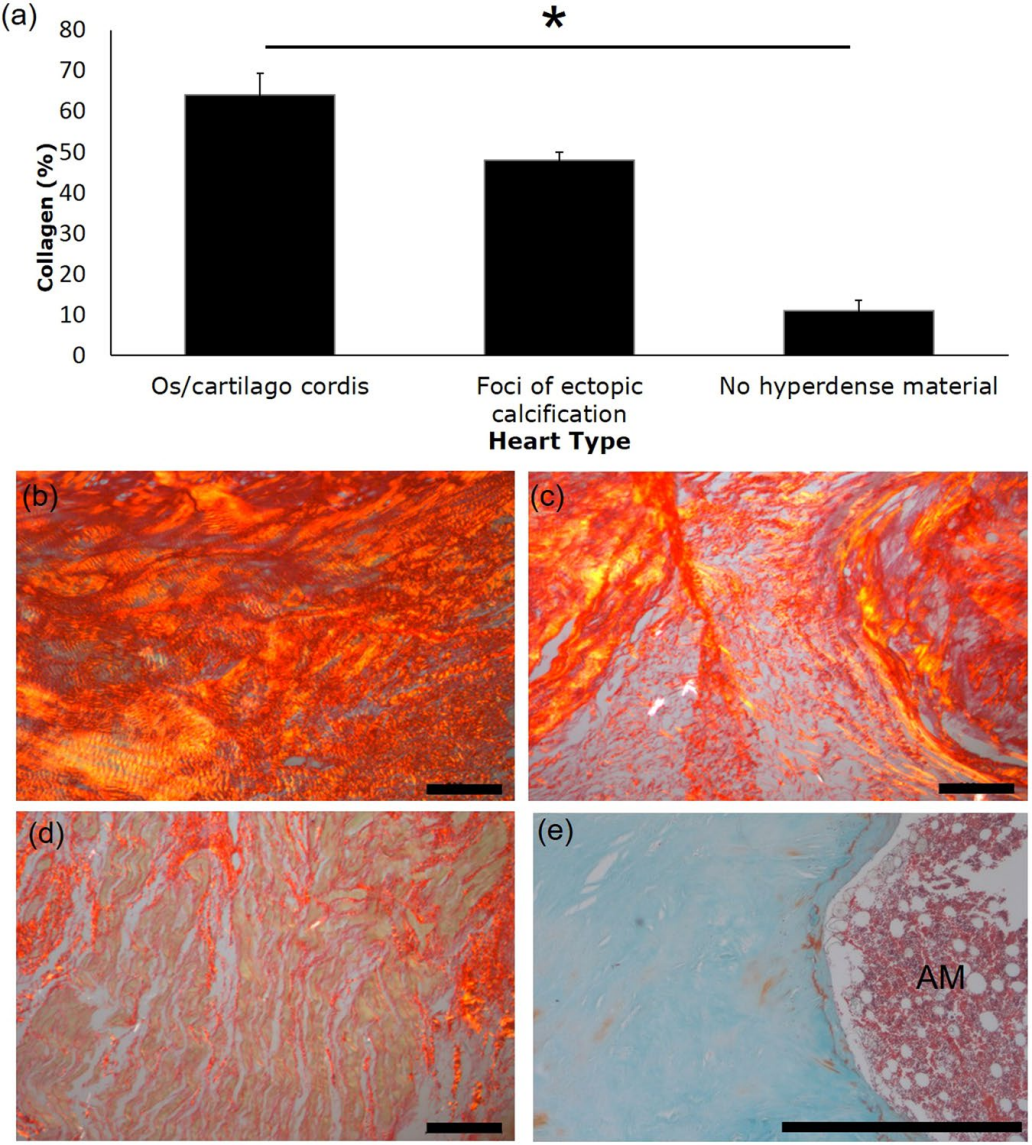
Collagen proportions in the cardiac skeleton. Graphical and photomicrograph representation of picrosirius stained tissue under polarised light showing the percentage of collagen present in the cardiac skeleton/myocardium tissue for areas surrounding bone/cartilage (n = 4; a,b), adjacent to foci of ectopic calcification (n = 2; a,c) and in myocardial tissue without hyperdense material (n = 4; a,d). Masson-trichrome staining showing tissue adjacent to bone containing adipose marrow (AM). Bars represent mean ± standard error of the mean. * Indicates P < 0.05. Scale bars represent 200 µm.

The percentage of collagen within the cardiac skeleton was increased in areas immediately adjacent to the os cordis or cartilago cordis in comparison to areas of the heart where neither bone, cartilage nor foci of ectopic calcification were present (mean ± standard deviation was 64.11 ± 10.61% around bone/cartilage compared to 10.91 ± 5.18% in tissue not immediately adjacent to bone/cartilage; P < 0.05; Fig. 6). A similar feature was observed around foci of ectopic calcification in comparison to tissue not containing hyperdense material but only two samples were present with these features therefore statistical analysis could not be carried out (47.90 ± 2.97% around foci of calcification compared to 10.91% in tissue not adjacent to hyperdense material; Fig. 6).

## Discussion

The aim of this study was to characterise the mineralised tissue discovered in the chimpanzee heart using microCT and histological techniques. This research has uncovered the presence of cartilage and/or bone formation in the cardiac skeleton of several chimpanzees. The bones and cartilage varied in size and two bones were trabeculated whilst the third was not. This is the first time that an os cordis has been observed in this species and also represents a rare observation in a non-bovid mammal. In addition, the relation between the presence of os cordis, cartilago cordis or ectopic calcifications and the level of IMF, age, sex of the animals and heart weight were studied. The significant association between the presence of an os cordis and high levels of IMF suggests that the presence of an os cordis in this species may be a pathological finding or marker rather than an anatomical feature.

In humans, cardiac CT is performed generally with the use of contrast agents in order to assess the morphology and function of the heart and great vessels, while non-contrast CT is mostly used for the detection and quantification of coronary artery calcification^35^. In this study, the use of microCT without contrast agents did not allow for the detection of IMF probably due to the relatively low differential attenuation of X-rays in biological tissue and potentially due to tissue fixation. It is probable that extra deposition of tissue of the same approximate density (rather than calcified) would be difficult to detect other than by measuring volume increases or thickening of the walls given standards to control against and assuming that the increases were large enough and did not simply replace existing tissues. Iodine staining techniques have been used elsewhere to enhance contrast for post-mortem cardiac tissue computed tomography^36,37^. Areas of mineralisation and ossification, however, were easily detected thus demonstrating that post-mortem microCT is a suitable technique for the detection of mineralised areas within the heart.

In humans, the coronary artery calcification score measurable by CT increases as a function of age in both men and women^35^, and deposition of calcium on the aortic cusps and on the fibrous skeleton of the base of the heart (mitral and aortic annuli) is also associated with aging^34^. Vascular calcification in humans is localised either in the tunica media or in the tunica intima of the vessel. Intima calcification, which was not observed in the chimpanzees in this study, is associated with atherosclerosis and formation of atherosclerotic plaques, whereas media calcification of larger vessels is more generalised and often found in the elderly or patients with chronic renal disease, hypertension and osteoporosis^38^. According to the available clinical history of the animals in the present study, S13, S14 and S16 showed evidence of chronic renal disease and S15 had end stage renal disease on post-mortem examination, which may explain the vascular media calcifications (ectopic calcifications) seen in their hearts. Chimpanzees are not as prone to coronary artery disease as humans^6^; however as they age, an increase in media calcifications was evident in our specimens, similar to findings in humans^39^. The microCT scan of the oldest chimpanzee in the present study, a female aged 59 years-old, revealed multiple areas of calcification within the great vessels. Pathological, histological and microCT results showed that she presented with only mild to moderate (level 3) IMF and no ossification nor cartilage in the cardiac skeleton, despite the multiple areas of ectopic calcification and her advanced age.

As three out of 16 examined hearts presented with bone formation within the trigonum fibrosum, it cannot be ruled out that the os cordis could be an anatomical peculiarity in some individuals of this species rather than linked to cardiovascular disease. Similarly, one heart contained cartilage within the trigonum fibrosum, a cartilago cordis. This mineralised cartilage area may be a precursor to bone formation in this animal. The exact function of cartilago or os cordis is unclear in other animal species. In cattle, the os cordis is believed to support normal motion of the valves in a heavy heart^27^ and has not been linked to cardiovascular disease. In otters (*lutra lutra)*, a smaller species, the presence of bone within the cardiac skeleton is also considered normal and its prevalence increases with age, as it was found in 11 out of 13 adult otter hearts but not in juveniles^29^. The presence of hyaline cartilage has also been described in the aorticopulmonary septum of individuals of eleven species of snakes, with a great variation in size, shape and precise location, and seemingly little functional influence^31^. In Syrian hamsters, the cartilaginous foci in the central fibrous body of the heart have been proposed to act as pivots resisting mechanical tensions generated during the cardiac cycle^32^.

In other species, however, bone and cartilage formation in the heart has been associated with CVD. In large breed dogs, although the presence of cartilage and bone formation in the central fibrous body of the heart has been reported as a normal occurrence at all ages, most of the dogs presenting with it are Doberman Pinschers, that suffer from a very high prevalence of dilated cardiomyopathy^40,41^. Moreover, some researchers have linked cardiac bone formation in dogs to sudden death, suggesting that the cartilage and bone formation relates to chronic ischemia, as they observed that the local small coronary arteries were normal in the os cordis area of cattle but were focally narrowed in the Dobermans^42^. A crossbred heavy horse with systemic circulatory disturbance presented with ectopic ossification with haematopoietic bone marrow in the heart valves and cardiac skeleton^43^. Degeneration and fibrosis of the atrioventricular nodal tissue and left bundle branch, associated with cartilage or bone in the central fibrous body, were observed in 63 cats with cardiomyopathy^44^. Finally, in humans, the presence of cartilage in the central fibrous body of the heart in relation to the conducting system was observed in two children who died suddenly^45^. Naturally it is still difficult to detach cause and correlation both in the above studies and our present findings linking os cordis and cartilago cordis to increased IMF levels.

Considering that the atrioventricular conducting system (Bundle of His) is the only electrical connection between the atria and the ventricles in a normal heart, it is possible that lesions or anatomical peculiarities affecting this area could result in altered function. One study evaluating the fibrous skeleton of the heart with CT and MRI imaging stated that large calcifications involving the central fibrous body can cause heart block by interfering with the normal function of the His bundle and its branches, and concludes that CT is the preferred technique for showing the extent of calcifications in the fibrous skeleton^46^.

In our study, despite the limited number of samples, the significant association between the presence of cartilage and/or bone and the level of IMF supports the theory that endochondral ossification occurs in areas of high mechanical forces and/or ischaemia. In humans, a certain degree of interstitial cardiac fibrosis is an age related-change that can lead to functional decline^47^ but is also seen secondary to pressure or volume overload and after myocardial infarction^10^. In chimpanzees, the pathophysiology of IMF is still under debate, as lesions can appear in animals as young as 10 years old^8^. Mineralised lesions, cartilage and bone formation are usually formed following a dystrophic calcification/mineralisation process, which generally occurs after an insult of the affected area resulting in necrosis. It is known that oxygen tension, pH, micronutrients and mechanical stimuli impacts bone formation. Hypoxic environment and an abnormally heightened or prolonged inflammatory response to injury are believed to be key factors in the developments of heterotopic ossifications in humans^48^. One possible theory based on our study is that in chimpanzees affected by IMF, chronic strain on the heart (as a result and/or cause of IMF) could also lead to heterotopic ossification of the cardiac skeleton, which is considered the fibrous structural support of the heart and an area exposed to high haemodynamic stress.

A novel finding was that the higher amounts of collagen present in the cardiovascular tissue (cardiac skeleton) surrounding areas of hyperdense material such as bone and cartilage. It is possible that collagen amounts were higher prior to the formation of hyperdense material, and/or could have developed after the hyperdense material formed, but it is difficult to measure in practical terms over time. It has been hypothesised that fibrous tissue is a prerequisite for the formation of cartilage and subsequently bone within the heart skeleton^29^. Normal foetal endochondral bone formation requires deposition of type I collagen followed by type II collagen at the onset of chondrocyte differentiation^49^. The cartilage becomes calcified and is replaced by osteoblasts depositing type I collagen once again^50^. This is in accordance with the results obtained in the present study, with evidence of endochondral ossification occurring within areas of mineralised cartilaginous metaplasia and expression of type I collagen surrounding areas of bone and no collagen III present. The bone formation observed in this study can be considered to be heterotopic ossification, in which bone forms in soft tissue structures within the body^51^. Less is understood about the role of collagen in heterotopic ossification, however one human study demonstrated that the collagen expression pattern in heterotopic ossification was largely similar to foetal bone formation^50^. As such it would be reasonable to assume that collagen deposition may be an important factor in the endochondral formation of bone in the cardiac skeleton of the chimpanzee. It has also been suggested that collagen synthesis can be upregulated due to mechanical stress on the heart^52,53^. This is in accordance with the theory that increased cardiac strain due to IMF is potentially leading to further fibrous tissue deposition and subsequent endochondral ossification.

The clinical and functional implications of the presence of cartilage and bone tissue in the chimpanzee cardiac skeleton remain to be elucidated. The ectopic vascular calcifications found in some chimpanzees appear to correlate well with the media calcifications reported in humans with chronic renal disease, hypertension and age. However, of the animals who presented with focal ossifications or cartilage formation in the cardiac skeleton, one died of sudden cardiac death and another following anaesthesia a few days after an episode of syncope. Myocardial fibrosis is known as a favourable substrate for the generation of re-entrant ventricular arrhythmias^54^, but whether the presence of cartilage formation or ossification within the cardiac skeleton of chimpanzees further increases the chances of arrhythmic events is, as yet, unknown.

Both IMF and foci of ectopic calcification and ossification within the heart are difficult to detect using conventional echocardiography and radiography; however, the relative ease of detection of both bone and mineralised cartilage by microCT in our specimens make these structures good candidates as ante-mortem markers of IMF in chimpanzees. However, it should be noted that not all chimpanzees affected with IMF had os/cartilago cordis, therefore it may serve as an indicator rather than diagnosis tool. The possibility of os cordis and cartilago cordis occurring in humans suffering from similar cardiovascular disorders should be considered. In conclusion this new discovery of both os cordis and a cartilago cordis, in the chimpanzee heart highlights the need for further cardiovascular investigations in this and other species including humans using the latest technologies in order to gain valuable clinical and anatomical knowledge.

## Methods

Formalin fixed whole hearts from chimpanzees that died in European zoos were received for detailed macroscopical and histopathological examination as part of the Ape Heart Project (led by Twycross Zoo and the University of Nottingham). The manner and cause of death were retrieved from the zoos’ submission forms and post-mortem records, together with other information such as sex, age and presence of significant comorbidities (see Supplementary Table 1). Ethical permission was given by The University of Nottingham, School of Veterinary Medicine and Science ethics committee in adherence to institutional and national guidelines (ethics number 1843 160905). Permission from each zoo was given for investigation of every animal and no chimpanzees were euthanised for the purposes of research.

### Heart macroscopical examination and histology

Examinations were carried out as per published protocols, similar to those carried out in cases of sudden cardiac death in humans^55–57^. Hearts were received fixed in neutrally buffered 10% formalin, with an incision made transversally across both ventricles approximately 3 cm from the apex to allow better fixation. Each heart was weighed, measured (length and circumference), the coronary artery sinuses were probed, and coronary arteries were cut at 3 mm intervals to check for dilatation or other vascular changes. The left and right ventricular walls as well as the interventricular septum were measured at the level of the transversal section. The transverse myocardium was assessed for any evidence of mottling or infarction. Major vessels and valves were examined, and valve circumferences were measured. A minimum of 12 samples were taken for histopathology, including three sections from the left ventricle, three sections from the right ventricle, two sections from the interventricular wall, and sections of the right ventricular outflow tract, the sino-atrial node region, the atrioventricular node region, and the aorta. Any abnormalities or lesions identified were also sampled.

Serial sections (7 µm per section) were cut and stained with haematoxylin and eosin and evaluated under the microscope by a diplomate from the European College of Veterinary Pathologists with expertise in great ape cardiovascular pathology. Pathological features such as myocyte degeneration, interstitial myocardial fibrosis, replacement fibrosis, perivascular fibrosis, cellular infiltration, myofibre disarray, myofibre necrosis, myofibre hypertrophy, vascular and valvular changes were assessed. Severity, location, distribution and chronicity of findings were described. Levels of IMF were ranked as follow: no IMF = level 0, minimal IMF = level 1, mild IMF = level 2, mild to moderate IMF = level 3, moderate IMF = level 4, moderate to marked IMF = level 5, marked IMF = level 6. Assigned IMF levels reflected the average levels of replacement, perivascular and interstitial fibrosis of all 12 slides. Every sample totalling eight hearts from female and eight hearts from male chimpanzees, presenting with different levels of IMF, and from individuals that died at different ages underwent microCT analysis (Table 1 and Supplementary Table 1).

### X-ray microtomography

Following pathological examination, each heart was wrapped in thin sheets of X-ray transparent polyethylene packing foam and placed into plastic specimen jars. A cone beam X-ray microCT scanner was used: Phoenix v|tome|x m (GE Sensing and Inspection Technologies GmbH, Wunstorf, Germany), set at 125 kV and 320 µA. The distances between the X-ray source and the sample and the X-ray source and the detector were optimised in order to achieve appropriate magnification and spatial resolution on a sample by sample basis. Some of the hearts were larger than others so the resolution was coarser. Each scan acquired over 2160 projection images over a 360° rotation of the sample using a detector exposure time of 333 ms, integrated over three averaged images, resulting in a total scan time of 48 min to 60 min depending on the heart size. Data were reconstructed using an inline median smoothing filter in datos|x software (GE Sensing and Inspection Technologies, Wunstorf, Germany), and exported as a 3D volume file. Higher resolution scans (6–10 micron range depending on sample size) were conducted on dissected regions to reveal the microstructure of the dense objects. High resolution scans were acquired at 80 kV, 120 µA, 200 ms detector timing and 2160 projection images with each image being the integration of 5 images to reduce noise. X-ray CT image data was visualised using VGStudioMAX v2.2 Software (Volume Graphics GmbH, Heidelberg, Germany). The high-density objects were digitally segmented from the 3D volumetric data based on their higher X-ray attenuation values in the images (brighter colour) and exported as image stacks. Mean local thickness for each object was measured using the BoneJ plugin for the open source image quantification and analysis software ImageJ 1.44^58,59^ following a protocol previously published^15^. Local thickness heat map images were imported and visualised in VGStudioMax.

### Hyperdense areas dissection and histology

Following pathology investigations and microCT analysis, areas containing hyperdense structures were dissected out of each heart and prepared for histopathology analysis (n = 4 samples containing bone/cartilage, n = 4 with no mineralised tissue present, n = 2 samples containing foci of ectopic calcifications). In brief, samples were processed through graded ethanol and xylene, prior to being embedded in paraffin. Serial sections (7 µm per section) were cut throughout each piece of tissue. A minimum of 8 sections per sample were then stained with haematoxylin and eosin. In addition, specimens with either bone, cartilage or foci of ectopic calcifications were stained with Masson-trichrome (100485; Merck KGaA, Germany) and Picrosirius red (ab150681; Abcam, UK) in order to quantitate total collagen levels immediately adjacent to the bone or cartilage. These specimens also underwent Von Kossa staining (ab150687; Abcam, UK) in order to evidence calcium deposition. Manual analysis of photomicrographs taken at 5×, 10×, 20X and 40X magnifications was carried out on all samples alongside the use of Image Pro (Media Cybernetics, USA) colour detection software of 5 images per section using systematic random sampling, both adjacent to the bone, cartilage or foci of ectopic calcification tissue and at 1 mm away from these areas in order to calculate the proportions of collagen^60^.

### Statistical analysis

Statistical analysis was conducted in IBM SPSS statistics 26. P-values less than 0.05 were considered statistically significant. A Shapiro-Wilk test revealed no significant deviation from normality for age (p = 0.522), heart weight (p = 0.416) but significant deviation from normality for IMF levels (p = 0.035). Thus, non-parametric tests were used when comparing levels of IMF with other variables. Independent samples Mann-Whitney U tests were used to compare levels of IMF with presence of bone/cartilage, ectopic calcifications, and sex. Exact significance levels were used for Mann-Whitney tests because of the small sample size. Spearman’s correlations were used to compare levels of IMF with age and heart weight. Independent samples T-tests were used to compare the presence of os cordis with age and heart weight, and to compare presence of vascular calcifications with age. Fisher’s exact tests were used to compare sex with presence of os cordis and ectopic calcifications (Fisher’s tests were used rather than Chi square tests because the expected values in the contingency tables were <5). Collagen proportions were compared using T-test (n = 4/group).

## Supplementary information

Supplemental information.

Supplemental information 2.
