# Supplementary material for: Discovery of os cordis in the cardiac skeleton of chimpanzees (Pan troglodytes)
Source: Sci Rep. 2020 Jun 10;10:9417. doi: 10.1038/s41598-020-66345-7 (PMC7286900; doi:10.1038/s41598-020-66345-7)
Supplement: Supplementary file 1 — Supplemental information. [file 41598_2020_66345_MOESM1_ESM.pdf]

Discovery of os cordis in the cardiac skeleton of chimpanzees (Pan troglodytes).

Sophie Moittié, Kerstin Baiker, Victoria Strong, Emma Cousins, Kate White, Mátyás Liptovszky, Sharon Redrobe, Aziza Alibhai, Craig J Sturrock and Catrin Sian Rutland

Supplementary Table 1

Chimpanzee sex, age, cause of death/comorbidities and other cardiac pathological findings.

Specimen ID	Sex	Age (yrs)	Cause of death +/- comorbidities	Other cardiac pathological findings
**S1**	Male	10	Accidental	None
**S2**	Male	11	Sudden death	None
**S3**	Male	19	Peri-anaesthetic	None
**S4**	Male	20	Peri-anaesthetic	None
**S5**	Male	22	Sudden death	Multifocal, mild, random areas of acute contraction band necroses
**S6**	Male	22	Died during anaesthesia for chronic air-sacculitis treatment	Multifocal, septic, coronary thrombi
**S7**	Male	28	Suspected tetanus, euthanised	Mild LVH
**S8**	Male	37	During anaesthesia a few days after a collapse	Moderate LVH and acute multifocal myocardial necroses
**S9**	Female	21	Euthanised (undisclosed reasons)	None
**S10**	Female	25	Systemic infection	Moderate LHV. Pancarditis and multiple septic thrombi
**S11**	Female	32	Accidental	Multifocal, mild, acute myocardial necroses
**S12**	Female	32	Euthanised due to uncontrollable diabetes	Multifocal, acute contraction band necroses
**S13**	Female	42	Sudden death. Evidence of chronic renal disease.	Moderate LVH
**S14**	Female	46	Euthanised due to hepatitis and sepsis. Evidence of chronic renal disease.	Multifocal, septic, coronary thrombi and multifocal, acute myocardial necroses
**S15**	Female	47	Euthanised due to end-stage renal disease	HCM
**S16**	Female	59	Euthanised due to multiple age-related disorders. Evidence of chronic renal disease	Focally extensive chronic infarction LV. Mild TV endocardiosis. Multifocal mild MV calcification
